# Hesperidin reduces depressive symptoms in post‐coronary artery bypass graft patients with mild depression

**DOI:** 10.1002/fsn3.3692

**Published:** 2023-09-28

**Authors:** Atie Sadat Khorasanian, Shima Jazayeri, Negar Omidi, Zahra Booyani, Mehrnaz Morvaridi, Mehdi Tehrani‐Doost, Agha Fateme Hoseini, Mostafa Nejatian, Naheed Aryaeian

**Affiliations:** ^1^ Department of Nutrition, School of Public Health Iran University of Medical Sciences Tehran Iran; ^2^ Research Center for Prevention of Cardiovascular Disease Institute of Endocrinology & Metabolism, Iran University of Medical Sciences Tehran Iran; ^3^ Department of Cardiology, Tehran Heart Center Tehran University of Medical Sciences Tehran Iran; ^4^ Department of Research Center for Cognitive and Behavioral Sciences Tehran University of Medical Sciences Tehran Iran; ^5^ Department of Biostatistics, School of Health Iran University of Medical Sciences Tehran Iran; ^6^ Department of Cardiac Rehabilitation, Tehran Heart Center Tehran University of Medical Sciences Tehran Iran

**Keywords:** brain‐derived neurotrophic factor, coronary artery bypass graft, cortisol, depression, hesperidin

## Abstract

Previous studies have shown that hesperidin may have beneficial effects on depression; however, to the best of our knowledge, no clinical trial has yet been conducted in this area. The aim of the present study was, therefore, to determine the effects of hesperidin on depression, serum brain‐derived neurotrophic factor (BDNF), and serum cortisol levels in post‐coronary artery bypass graft (CABG) patients. Toward this goal, 73 post‐CABG patients with depression symptoms were enrolled. The participants were randomly divided into two groups to receive either 200 mg/day hesperidin (*n* = 38) or placebo (*n* = 35) for 12 weeks. Depressive symptoms, serum BDNF, and cortisol levels were then assessed at the baseline and after intervention. Beck Depression Inventory‐II (BDI‐II) was also used to determine the severity of depression. Sixty‐six patients completed the trial. Hesperidin decreased depression severity after 12 weeks, as compared to placebo (*p* = .004), but serum BDNF and cortisol were not statistically significantly different in the two groups after the intervention. Subgroup analyses also showed that, while in the patients with mild depression, the score of BDI‐II was significantly different in the hesperidin and placebo groups after intervention; there was no difference in the severity of depression between the two groups in patients with moderate‐to‐severe depression. To conclude, a dose of 200 mg/day hesperidin may reduce depressive symptoms after 12 weeks in post‐CABG patients with mild depression.

## Graphical abstract

The aim of the present study was to determine the effects of hesperidin on depression, serum brain‐derived neurotrophic factor (BDNF), and serum cortisol levels in post‐coronary artery bypass graft (CABG) patients. The participants were randomly divided into two groups to receive either 200 mg/day hesperidin (*n* = 38) or placebo (*n* = 35) for 12 weeks. A dose of 200 mg/day hesperidin can reduce depressive symptoms after 12 weeks in post‐CABG patients with mild depression.

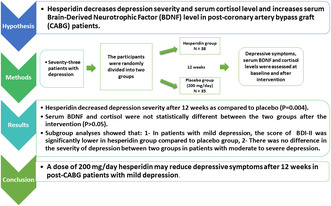

## 1 INTRODUCTION

Depression can affect a person's thoughts, behavior, feelings, and sense of well‐being, and people with a depressed mood can feel sad, anxious, absurd, hopeless, helpless, worthless, guilty, irritable, angry, embarrassed, or restless (Association, 2013; Salmans, 1995). Depression is common after coronary artery bypass graft (CABG) surgery, being a strong independent predictive factor for cardiac morbidity and mortality (Borowicz et al., 2002; Connerney et al., 2001; Pietrzyk et al., 2014; Rothenhäusler et al., 2005). Thirty to forty percent of patients who undergo CABG experience depression (Stenman et al., 2022). It can lead to complications such as increased re‐hospitalization, heart failure, stroke, myocardial infraction, and poor physical and emotional recovery (Goudarzi et al., 2023; Goyal et al., 2005; Morone et al., 2010; Oxlad et al., 2006). Depression also has a major impact on the quality of life of people with cardiovascular diseases, increasing their risk of cardiac readmission up to 6 months after surgery and the rate of cardiac events up to 8 years (AbuRuz & Al‐Dweik, 2022; Stenman et al., 2022).

Hesperidin (4′‐methoxy‐7‐O‐rutinosyl‐3′,5‐dihydroxyflavanone) is a glycosylated flavonoid found abundantly in citrus fruits, especially oranges, mandarins, and lemons (Peterson et al., 2006; Yang et al., 2011); it can cross the blood–brain barrier (Dimpfel, 2006). Studies have shown that hesperidin exhibits various biological properties including antioxidant, anti‐inflammatory, anticancer and anti‐allergic, and neuroprotective effects (Garg et al., 2001; Thenmozhi et al., 2015). The obtained evidence has shown that hesperidin has a protective effect on cardiovascular diseases due to its anti‐inflammatory and antioxidant properties (Parhiz et al., 2015). It has also been suggested that these antioxidants have beneficial effects in the treatment of myocardial toxicity through the activation of PPAR‐γ (Agrawal et al., 2014).

Animal studies have shown that hesperidin has antidepressant‐like effects through several mechanisms, such as inhibiting serotonergic 5‐HT_1A_ receptors and increasing serotonin and dopamine in hippocampus, cerebral cortex, and whole brain (Nadar et al., 2018; Souza et al., 2013). Some studies have also shown that the antidepressant‐like properties of hesperidin are exerted through their interaction with kappa‐opioid receptors and inhibition of the pathway of l‐arginine‐NO‐cGMP; ultimately, there is a decrease in nitrate or nitrite levels in the hippocampus by inhibiting potassium channels in the animal models of depression (Carlos Filho et al., 2013; Donato et al., 2014, 2015). Brain‐derived neurotrophic factor (BDNF) is a member of the family of neurotrophic growth factors. It has been demonstrated that serum or plasma BDNF levels are decreased in depressed patients, as compared to the healthy controls, and attenuated BDNF levels are boosted by antidepressants (Gonul et al., 2005; Jones & Reichardt, 1990; Yang et al., 2021). On the other hand, previous research suggests that hesperidin exhibits antidepressant‐like properties due to induced BDNF in the hippocampus (Donato et al., 2014; El‐Marasy et al., 2014; Kosari‐Nasab et al., 2018; Li et al., 2016). Moreover, antidepressant‐like effects of hesperidin may be mediated by regulating hypothalamus–pituitary–adrenal (HPA) axis, increasing the nerve growth factor (NGF), and regulating the levels of pro‐inflammatory cytokines such as IL‐1β and IL‐6 (Antunes et al., 2016; Cai et al., 2013; Donato et al., 2014; El‐Marasy et al., 2014; Fu et al., 2019). Another antidepressant‐like mechanism of hesperidin is reducing and, ultimately, regulating the activity of acetylcholinesterase activity, which is similar to the mechanism of the action of antidepressants (Antunes et al., 2016; Müller et al., 2002).

Although there is strong evidence, based on animal studies, that hesperidin may be effective in decreasing depression, so far, to the best of our knowledge, no clinical trial has been conducted in this area. Because of the relatively high prevalence of depressive symptoms in post‐CABG patients, the current study was conducted on this group of patients. The aim of the present study was, therefore, to determine the effects of hesperidin on the severity of depression, serum BDNF, and cortisol levels in post‐CABG patients.

## 2 METHODS

### 2.1 Trial design

The present study was a randomized double‐blind placebo‐controlled trial (RCT) conducted on post‐CABG patients. This study was conducted according to the guidelines laid down in the Declaration of Helsinki; all procedures involving human patients were approved by the Ethics Committee, Iran University of Medical Sciences, Tehran, Iran (IR.IUMS.REC 1395.9321323003). Written informed consent was obtained from all patients. This trial was registered in the Iranian Registry of Clinical Trials (www.irct.ir), with the identifier IRCT201702262394N36. Seventy‐three patients were recruited for the purpose of this study. The participants were divided into two groups to receive either 200 mg/day hesperidin or placebo for 12 weeks. The patients were allocated to two groups by permuted block randomization. To blind the study, hesperidin and placebo capsules were similar in appearance and the project managers and participants were fully ignorant (blind) of the intervention and control groups.

### 2.2 Participants

The patients were referred from the Department of Cardiology, Tehran Heart Center, Tehran, Iran, between August 2018 and February 2019. The inclusion criteria were the age range of 30–70 years, willingness to participate, patients undergoing isolated CABG within 3 months after myocardial infarction, a BDI‐II score ≥ 10, not having taken any dietary supplement in the past 3 months, not having used more than one red orange or two other citrus fruits per day, as well as a BMI = 25–40. The exclusion criteria were pregnancy and lactation, having psychiatric or neurological disorders, having allergy to any antioxidant and vitamin supplements, use of antidepressant drugs in the past 1 month, use of corticosteroids, smoking, use of heparin and warfarin, allergy symptoms at any time during the study, the occurrence of uncontrolled chronic illnesses, gastrointestinal disorders, unwillingness to continue the study, change in the dose of drugs used, such as anti‐lipid drugs, during the 3‐month study, ejection fraction <30%, and compliance <80%.

### 2.3 Intervention

Participants received either 200 mg/day hesperidin or placebo, orally, after lunch for 12 weeks. Hesperidin extracted from the *Citrus aurantium* was supplied by the Kripps Company, Canada, as 200 mg capsules. The selected dose was determined based on the previous studies (Constans et al., 2015; Perche et al., 2014). The placebo capsules containing 200 mg starch were produced by the Department of Pharmacokinetics, School of Pharmacy, Tehran University of Medical Sciences, Tehran, Iran.

### 2.4 Outcomes

The change in depression severity was the primary outcome measure assessed by the Beck Depression Inventory‐II (BDI‐II). Secondary outcomes were changes in the serum BDNF and cortisol.

### 2.5 Clinical measurements

Depression was assessed by validated Persian version of BDI‐II. BDI‐II consisted of 21 questions with four possible answers sorted according to symptom severity and score ranges from 0 to 63 points (Ghassemzadeh et al., 2005). The following ranges are usually used: minimal (0–9), mild (10–18), moderate (19–29), and severe depression (30–63) (Beck et al., 1961).

To control the confounding factors, dietary intake and physical activity were assessed by 3‐day 24‐h dietary recall and international physical activity questionnaires, respectively, at the baseline and after intervention. Moreover, weight was measured by Seca scale, and height was measured using stadiometer at the beginning of the study and after the 12‐week treatment. Compliance of the patients was assessed by counting pills.

### 2.6 Blood sampling and laboratory tests

At the baseline and after 12 weeks, blood samples were collected between 8 and 9 a.m. after 10–12 hours of fasting to measure the serum cortisol and BDNF levels. After centrifugation of the samples, the serum was separated and frozen at −80°C for the subsequent analysis. Serum BDNF concentration was measured using commercial enzyme‐linked immunosorbent assay (ELISA) kits, Shanghai crystal Day Biotech Co., LTD, China, with a sensitivity of 0.01 ng/mL. In addition, commercial ELISA kits with a sensitivity of 0.366 μg/dL were used to measure serum cortisol (Monobind Inc.).

### 2.7 Statistical analysis

Sample size calculation was performed for the primary outcome, assuming at least 80% power and an α of 0.05, which was based on the previous study with a standard deviation of 2.5 for the severity of depression (Haberka et al., 2013). SPSS, version 16, was used to analyze the obtained data. The patients were stratified into two groups according to the baseline BDI‐II score. Mild‐ or moderate‐to‐severe depression was defined as 10 ≤ baseline BDI‐II, score ≤ 18, and BDI‐II and score > 18 score, respectively. As the number of patients with severe depression was very low, we decided to combine them with the patients having moderate depression. The normality of data was assessed using the Kolmogorov–Smirnov test. The independent sample *t*‐test and Mann–Whitney test, depending on the normal distribution, were then conducted to assess the differences between the two groups. Paired t‐test and Wilcoxon test, depending on the normal distribution, were also used to compare the variables before and after intervention in either group. Analysis of covariance, adjusted for the difference in iron and EPA, and baseline BMI was done to compare the severity of depression, cortisol, and BDNF levels between groups after the intervention. The chi‐square test was also used for the categorical variables. A *p*‐value <.05 was considered to show the possible statistical significance.

## 3 RESULTS

**FIGURE 1:**
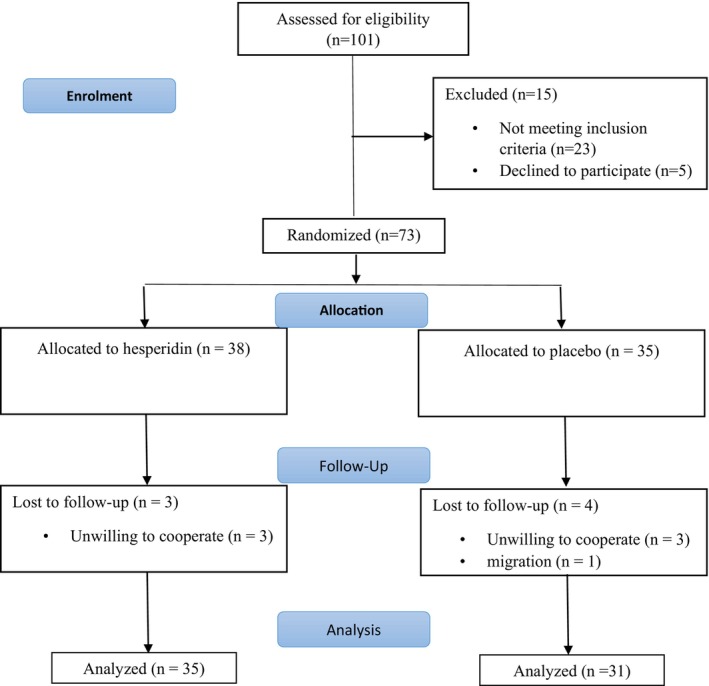
CONSORT flow diagram.

As shown in Figure 1, of the 73 patients recruited for the purpose of this study, 66 (35 patients in the hesperidin group and 31 in the placebo group) completed the study and were compliant with the protocol of the study. None of the patients reported significant adverse events during the trial.

According to Table 1, at the baseline, there was no significant difference between the two groups, except body mass index (BMI) (*p* = .047). With regard to dietary intakes and physical activity, there were no significant differences between the two groups at the baseline, but the intake of iron was increased (*p* = .043), while that of eicosapentaenoic acid (EPA) was decreased (*p* = .03) in the hesperidin group after intervention. However, adjusting for these variables in data analysis did not affect the results in regard to statistical significance.

**TABLE 1:** Baseline characteristics of the participants.

Variables	Hesperidin (*n* = 31)	Placebo (*n* = 35)	*p*‐Value^a^
Age (years) mean ± SD	59.77 ± 7.55	58.32 ± 7.17	.42^b^
Gender, *n* (%)			
Male	24 (68.6%)	24 (77.4%)	.42^c^
Female	11 (31.4%)	7 (22.6%)	
Weight (kg) mean ± SD	76.21 ± 12.1	75.57 ± 6.31	.79^b^
Height (cm) mean ± SD	165.65 ± 8.49	168.7 ± 8	.14^b^
BMI (kg/m^2^) median (Q1–Q3)	27.5(25.1–29.9)	25.4 (24.93–27.4)	.047^d^ ^,^ *
Previous history of depression, *n* (%)			
Yes	3 (8.6%)	4 (12.9%)	.59^c^
No	32 (91.4%)	27 (87.1%)	
Family history of depression, *n* (%)			
Yes	0	2 (6.5%)	.31^c^
No	35 (100%)	29 (93.5%)	
Time after CABG (month) mean ± SD	1.5 ± 0.5	1.5 ± 0.5	.14^b^

Abbreviations: BMI, body mass index; CABG, coronary artery bypass surgery graft; SD, standard deviation.

^a^ *p*‐Values for differences between two groups.

^b^ Independent sample *t*‐test.

^c^ Chi square test.

^d^ Mann–Whitney test.

* *p*‐Value of <.05 was considered significant.

The results related to depression severity are shown in Table 2. Among 66 patients who completed the study, hesperidin decreased the score of BDI‐II after 12 weeks, as compared to placebo (*p* = .004). Subgroup analysis also showed that, in patients with mild depression, the severity of depression was significantly different between hesperidin and placebo groups after intervention. Meanwhile, subgroup analysis did not show any difference in the score of BDI‐II between hesperidin and placebo groups in patients with moderate‐to‐severe depression.

**TABLE. 2:** BDI score at the baseline and after 12 weeks.

	Groups/*p*‐Value^a^	Baseline	After 12 weeks	*p*‐Value^b^
Patients with mild depression	Hesperidin (*n* = 12) mean ± SD	13.83 ± 2.12	8.08 ± 2.42	*p* < .001^c^ ^,^ *
	Placebo (*n* = 23) mean ± SD	12.47 ± 2.46	13.91 ± 4.77	.16^c^
	*p*‐Value	.12^d^	*p* < .001^e^ ^,^ *	
Patients with moderate‐to‐severe depression	Hesperidin (*n* = 23) mean ± SD	28.78 ± 9.2	15.26 ± 7.57	*p* < .001^c^ ^,^ *
	Placebo (*n* = 8) mean ± SD	24 ± 6.54	18.25 ± 7	.07^c^
	*p*‐Value	.18^d^	.32^e^	
Total patients	Hesperidin (*n* = 35) median (Q1–Q3)	22 (15–29)	12 (7–16)	*p* < .001^g^ ^,^ *
	Placebo (*n* = 31) median (Q1–Q3)	13 (11–19)	15 (11–19)	.95^g^
	*p*‐Value	*p* < .001^f^ ^,^ *	.004^e^ ^,^ *	

*Note*: Data are expressed as mean ± standard deviation or median (Q1–Q3).

^a^ *p*‐Values for differences between two groups.

^b^ *p*‐Values for differences within the group.

^c^ Paired sample *t*‐test.

^d^ Independent sample *t*‐test.

^e^ Analysis of covariance adjusted for the difference in iron and EPA, baseline BMI, and baseline BDI‐II score.

^f^ Mann–Whitney test.

^g^ Wilcoxon test.

* *p*‐Value of <.05 was considered significant.

Data were analyzed again by the inclusion of all participants according to their treatment assignments (data not shown). The results of an intention‐to‐treat analysis (in the 73 patients who were registered for the study) were compared with those of a pre‐protocol; similar results were obtained in the intention‐to‐treat population. As shown in Table 3, serum BDNF and cortisol were not significantly different in the two groups after intervention (*p* > .05).

**TABLE. 3:** Serum BDNF and cortisol at the baseline and after 12 weeks.

	Variables	Groups/*p*‐value^a^	Baseline	After 12 weeks	*p*‐Value^b^
Patients with mild depression	BDNF (ng/mL)	Hesperidin (*n* = 12)	0.47 (0.37–0.68)	0.65 (0.53–0.75)	.02^e^ ^,^ *
		Placebo (*n* = 23)	0.46 (0.37–0.61)	0.64 (0.53–0.9)	.06^e^
		*p*‐Value	.86^d^	.88^f^	
	Cortisol (μg/dL)	Hesperidin (*n* = 12)	14.95 (11.97–17.27)	17.6 (12.55–19.5)	.14^c^
		Placebo (*n* = 23)	18.70 (16.5–21.7)	16.7 (14.1–18.6)	.29^e^
		*p*‐Value	.007^d^ ^,^ *	.70^f^	
Patients with moderate‐to‐severe depression	BDNF (ng/mL)	Hesperidin (*n* = 23)	0.47 (0.36–1)	0.76 (0.57–1.16)	.008^e^ ^,^ *
		Placebo (*n* = 8)	0.50 (0.34–0.56)	0.68 (0.5–0.84)	.16^e^
		*p*‐Value	.83^d^	.97^f^	
	Cortisol (μg/dL)	Hesperidin (*n* = 23)	15.9 (12.1–18)	16 (12.8–18.4)	.46^e^
		Placebo (*n* = 8)	17.8 (16.65–24.65)	15.95 (15.05–18.42)	.23^e^
		*p*‐Value	.06^d^	.45^f^	
Total patients	BDNF (ng/mL)	Hesperidin (*n* = 35)	0.47 (0.37–0.94)	0.69 (0.57–0.92)	.001^e^ ^,^ *
		Placebo (*n* = 31)	0.47 (0.37–0.6)	0.66 (0.53–0.86)	.02^e^ ^,^ *
		*p*‐Value	.77^d^	.121^f^	
	Cortisol (μg/dL)	Hesperidin (*n* = 35)	15.9 (12.1–17.9)	16.3 (12.8–19.2)	.81^e^
		Placebo (*n* = 31)	18.6 (16.6–22.1)	16.6 (14.8–18.6)	.13^e^
		*p*‐Value	.001^d^ ^,^ *	.203^f^	

*Note*: Data are expressed as median (Q1–Q3).

^a^ *p*‐Values for differences between two groups.

^b^ *p*‐Values for differences within the group.

^c^ Paired sample *t*‐test.

^d^ Mann–Whitney test.

^e^ Wilcoxon test.

^f^ Analysis of covariance adjusted for the difference in Iron and EPA, baseline BMI, and baseline values.

* *p*‐Value of <.05 was considered significant.

## 4 DISCUSSION

In post‐CABG patients with mild depression, hesperidin at a dose of 200 mg/day for 12 weeks reduced the severity of depression, as compared to placebo, but it had no effect on serum BDNF and cortisol levels. In patients with moderate‐to‐severe depression, although the severity of depression was decreased in the hesperidin group, the differences in the score of BDI‐II and other variables between the two groups were not statistically significant.

Many studies have shown that hesperidin has antidepressant‐like effects in animal models of depression (Antunes et al., 2016; Cai et al., 2013; Kosari‐Nasab et al., 2018; Li et al., 2016). Furthermore, antidepressant‐like effects of hesperidin have been compared with standard antidepressants, such as imipramine and fluoxetine; it has been concluded that hesperidin could be as effective as antidepressants in reducing depressive behaviors (Antunes et al., 2016; Cai et al., 2013; Fu et al., 2019; Kosari‐Nasab et al., 2018; Li et al., 2016). Consistent with the findings of animal studies, the results of our study showed that hesperidin could reduce the severity of depression in patients with mild depression. So, to the best of our knowledge, no clinical trial has been conducted in this area, but a limited number of clinical trials have investigated the effects of other phytochemicals such as curcumin, resveratrol, and crocin on depression (Jam et al., 2017; Kanchanatawan et al., 2018; Lopresti, 2017; Malaguarnera et al., 2018). A review of animal and human studies by Lopresti also concluded that curcumin might be an effective antidepressant for patients with major depressive disorder (Lopresti, 2017). However, the results of studies included in this review were not consistent. In addition, another RCT in patients with depression and mean age of 42.6 years showed that 1500 mg curcumin (250 mg curcumin capsules for oral administration twice daily; they were asked to increase the dose every week) after 12 weeks had a significant antidepressant effect, as compared to placebo (Kanchanatawan et al., 2018). Moreover, taking 19.8 mg of resveratrol in oral pills for 90 days in subjects aged 39 and 60 with moderate depression and 15 mg crocin in oral tablets twice daily for 8 weeks in subjects with a mean age of 48 years and mild depression reduced the severity of symptoms. Both studies also included placebo groups (Jam et al., 2017; Malaguarnera et al., 2018).

Animal studies have shown that the antidepressant‐like effect of hesperidin is mediated through interaction with 5‐HT_1A_ (Souza et al., 2013) and kappa‐opioid serotonergic receptors (Carlos Filho et al., 2013). Marasy et al. have shown that hesperidin exerts its antidepressant effect through antioxidant and anti‐inflammatory properties, increased neurogenesis, and changes in monoamine levels in the brain (El‐Marasy et al., 2014). On the other hand, some evidence suggests that the antidepressant‐like effect of hesperidin may be exerted by inhibiting potassium channels (Donato et al., 2015) and blocking the l‐arginine‐NO‐cGMP pathway, subsequently reducing nitrate or nitrite levels in the rat hippocampus (Donato et al., 2014). In addition, hesperidin is involved in maintaining brain plasticity and regulating acetylcholinesterase activity, which is very similar to the mechanism of the action of antidepressants (Antunes et al., 2016). In the present study, hesperidin significantly reduced the severity of depression in subjects with mild depression, but the effect of hesperidin on depression was not observed in the patients with moderate‐to‐severe depression, probably due to inadequate sample size, low dose, or duration of the intervention. On the other hand, it is possible that hesperidin alone could not affect patients with moderate‐to‐severe depression, but if these patients were taking antidepressants, hesperidin might have increased the effects of the drugs. A previous study has also shown that curcumin can increase the effects of antidepressants (escitalopram orally with daily doses ranging from 5 to 15 mg within 1 week) in patients with major depressive disorder (more‐severe form of depression) (Yu et al., 2015). There is a possibility that hesperidin could show its effect in combination with medications in patients with moderate‐to‐severe depression and that the use of hesperidin alone might not have a significant effect in this subgroup of patients.

Recent evidences have shown that neurogenesis by neurotrophic factors can prevent cellular atrophy and the loss of neurons in depression (Banasiak‐Cieślar et al., 2023; Banasr et al., 2011). BDNF is a member of the family of neurotrophic growth factors (Jones & Reichardt, 1990). It has been reported that serum BDNF levels are reduced in patients with depression, as compared to healthy subjects, and antidepressants might increase it (Gonul et al., 2005). Although, to our knowledge, no clinical trials have been conducted to investigate the effect of hesperidin on serum BDNF levels, animal studies have shown that hesperidin can increase BDNF in the hippocampus through increased extracellular signal‐regulated kinase (ERK) phosphorylation (Donato et al., 2014; El‐Marasy et al., 2014; Kosari‐Nasab et al., 2018; Li et al., 2016). In our study, BDNF level was not different in hesperidin and placebo groups after intervention. The number of clinical trials assessing the effect of phytochemicals on BDNF is very limited. In this regard, JJ Yu et al. showed that, compared to antidepressants alone, 1000 mg curcumin in oral capsule plus antidepressant (escitalopram) significantly increased plasma BDNF levels in 108 men with major depression after 6 weeks (Yu et al., 2015). In contrast to the findings of Yu et al., hesperidin did not affect BDNF in the present study, probably because the participants did not receive any antidepressant drugs. The hypothesis put forward in our study is that hesperidin might exert its antidepressant effects independently of BDNF. Hesperidin has powerful antioxidant and anti‐inflammatory properties. In addition to scavenging free radicals, it increases cellular antioxidant defenses through the ERK/Nrf2 signaling pathway. This flavonoid also exhibits anti‐inflammatory properties through its effects on the nuclear factor Κβ pathway. Both antioxidant and anti‐inflammatory properties have impact on neurogenesis, neuron activity, and monoamines in depression (Parhiz et al., 2015; Tejada et al., 2018). The antidepressant effect of hesperidin on BDNF was investigated in the hippocampus of animal models, and we studied the effect of hesperidin on serum BDNF. Possibly, hesperidin can only affect the BDNF hippocampus (or central nervous system) levels, not at systemic level. Small sample size can be another possible suggestion. Since the sample size was measured using the primary outcome, depression severity, it was possible that the number of patients was insufficient to show changes in serum BDNF.

Dysregulation of HPA axis has been implicated in the pathophysiology of depression (Pariante & Lightman, 2008). Although previous studies have shown that hesperidin can reduce the depressive behaviors of rats by regulating the HPA axis (Cai et al., 2013; Li et al., 2016), hesperidin did not affect cortisol in the present study. To our knowledge, no previous trial has assessed the effects of hesperidin on cortisol, but three RCTs have assessed the effects of other phytochemicals on cortisol (Cicero et al., 2020; Poulsen et al., 2013; Yu et al., 2015). In contrast to our findings, curcumin has been shown to significantly reduce serum and salivary cortisol concentrations in overweight pre‐diabetic patients (two oral tablets containing 800 mg phytosomal curcumin per day for 8 weeks) and patients with major depressive disorder (two oral capsules containing 1000 mg of curcumin powder daily for 6 weeks), respectively (Cicero et al., 2020; Yu et al., 2015). In these two studies, cortisol levels were higher than the normal range at the baseline. Similar to our study, serum cortisol levels were normal in the participants of Morten's study who were healthy obese men; thus, no effect of resveratrol on serum cortisol levels was reported (Poulsen et al., 2013). In addition, a recent study has shown that salivary evening cortisol, not morning cortisol, maybe more correlated with depressive symptoms (Høifødt et al., 2019). Therefore, in the present study, the lack of effect of hesperidin on serum cortisol levels after the intervention could be due to the measurement of serum cortisol levels in the morning.

The present study was the first double‐blind placebo‐controlled clinical trial assessing the effects of hesperidin on the severity of depression, serum cortisol, and BDNF levels in post‐CABG patients. The dose of hesperidin used in this study was approximately equivalent to two glasses of red orange juice; as 1 L of orange juice contains 200–600 mg hesperidin with the highest level in red orange (Erdman et al., 2007). Consuming 200 mL of blond orange juice three times a day for 4 weeks resulted in increased antioxidant activity and improved vascular function (Constans et al., 2015). However, our study faced some limitations. Hesperidin levels were not measured in the plasma of the participants. In addition, the activity of HPA axis was assessed by serum cortisol, not by the DEXA test; also, serum cortisol levels were measured in the morning. Moreover, some confounding factors such as the severity of pain and length of hospitalization or complications of surgery were not investigated.

## 5 CONCLUSIONS

To conclude, after 12 weeks, hesperidin at a dose of 200 mg/day may reduce depressive symptoms in post‐CABG patients with mild depression. However, it is recommended to conduct studies with larger sample sizes and investigate other probable mechanisms of the effects of hesperidin on depression.

## AUTHOR CONTRIBUTIONS

**Atie Sadat Khorasanian:** Conceptualization (equal); data curation (equal); funding acquisition (equal); investigation (equal); methodology (equal); project administration (equal); resources (equal); supervision (equal); writing – original draft (equal); writing – review and editing (equal). **Shima Jazayeri:** Data curation (equal); formal analysis (equal); methodology (equal); project administration (equal); validation (equal); visualization (equal); writing – review and editing (equal). **Negar Omidi:** Methodology (equal); visualization (equal); writing – review and editing (equal). **Zahra Booyani:** Data curation (equal); investigation (equal). **Mehrnaz Morvaridi:** Writing – review and editing (equal). **Mehdi Tehrani‐Doost:** Methodology (equal); project administration (equal); writing – review and editing (equal). **Agha Fateme Hoseini:** Formal analysis (equal); software (equal). **Mostafa Nejatian:** Data curation (equal). **Naheed Aryaeian:** Supervision (equal).

## FUNDING INFORMATION

This research was supported by a grant from the Iran University of Medical Sciences (95‐04‐27‐30284).

## CONFLICT OF INTEREST STATEMENT

All authors declared no conflict of interest.

## Data Availability

The data that support the findings of this study are available on request from the corresponding author. The data are not publicly available due to privacy or ethical restrictions.
